# Supplementary material for: Revealing the role of ionic liquids in promoting fuel cell catalysts reactivity and durability
Source: Nat Commun. 2022 Oct 26;13:6349. doi: 10.1038/s41467-022-33895-5 (PMC9606256; doi:10.1038/s41467-022-33895-5)
Supplement: Supplementary file 1 — Supplementary Info [file 41467_2022_33895_MOESM1_ESM.pdf]

Supplementary Information

Revealing the Role of Ionic Liquids in Promoting Fuel Cell Catalysts Reactivity and Durability

Arezoo Avid[a,b], Jesus López Ochoa[a,b], Ying Huang[b,c], Yuanchao Liu[a,b], Plamen Attanassov[a,b], Iryna V. Zenyuk[a,b*]

a. Department of Chemical and Biomolecular Engineering, University of California Irvine, 221 Engineering Service Rd., Irvine, CA, 92617

b. National Fuel Cell Research Center, University of California Irvine, 221 Engineering Service Rd., Irvine, CA, 92617

c. Department of Materials Science and Engineering, University of California Irvine, 221 Engineering Service Rd., Irvine, CA, 92617

*Corresponding Author: iryna.zenyuk@uci.edu

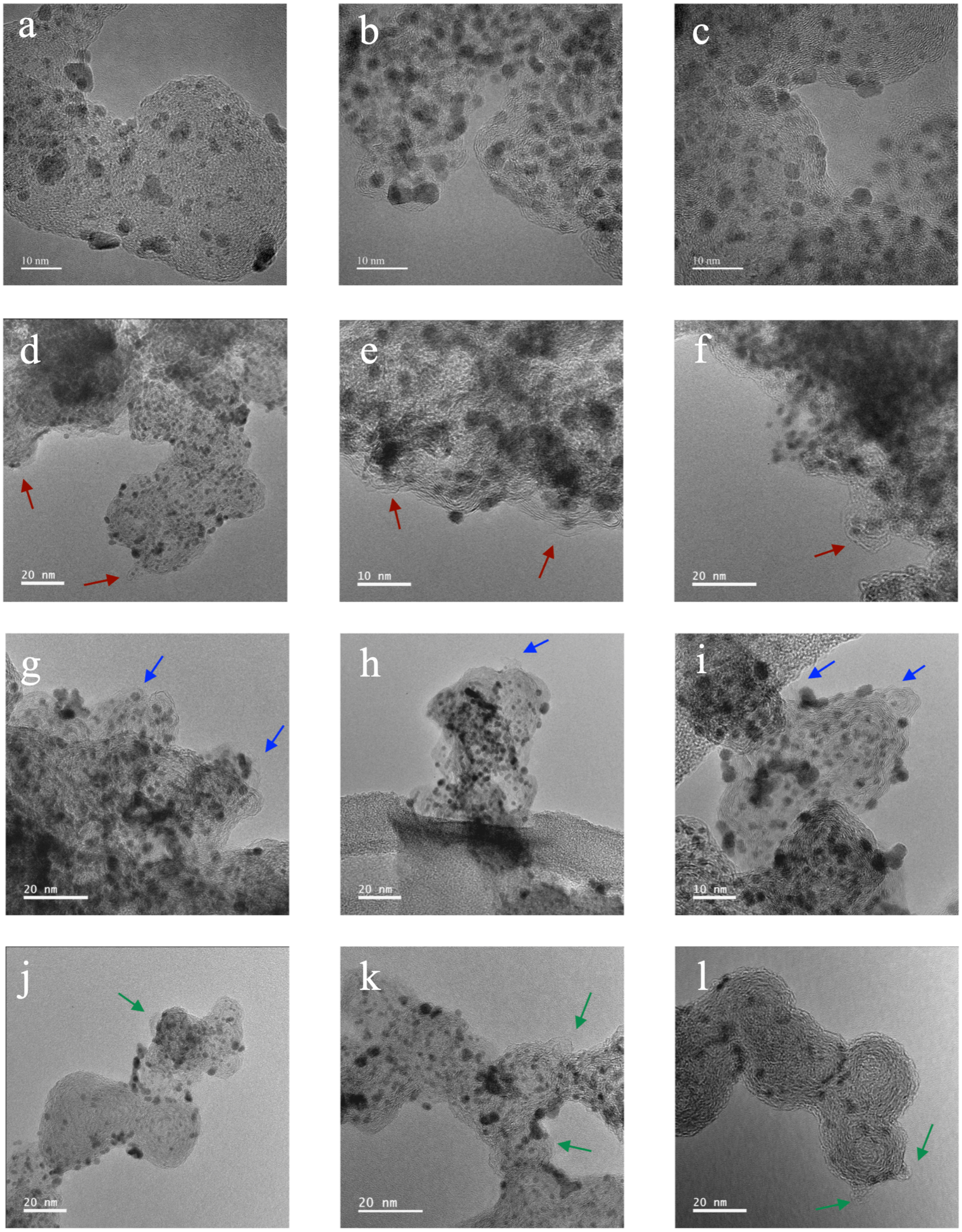

Figure S1. TEM images of a, b, c) Baseline (pristine) 40 wt. % Pt/C obtained from Fuel Cell Store, IL-modified Pt/C catalysts with d, e, f) Pt/C-([C_2mim]$^+$[NTf_2]$^-$), g, h, i) Pt/C-([C_4mim]$^+$[NTf_2]$^-$), and j, k, l) Pt/C-([C_4dmim]$^+$[NTf_2]$^-$). Arrows show the ionic liquids lumps.

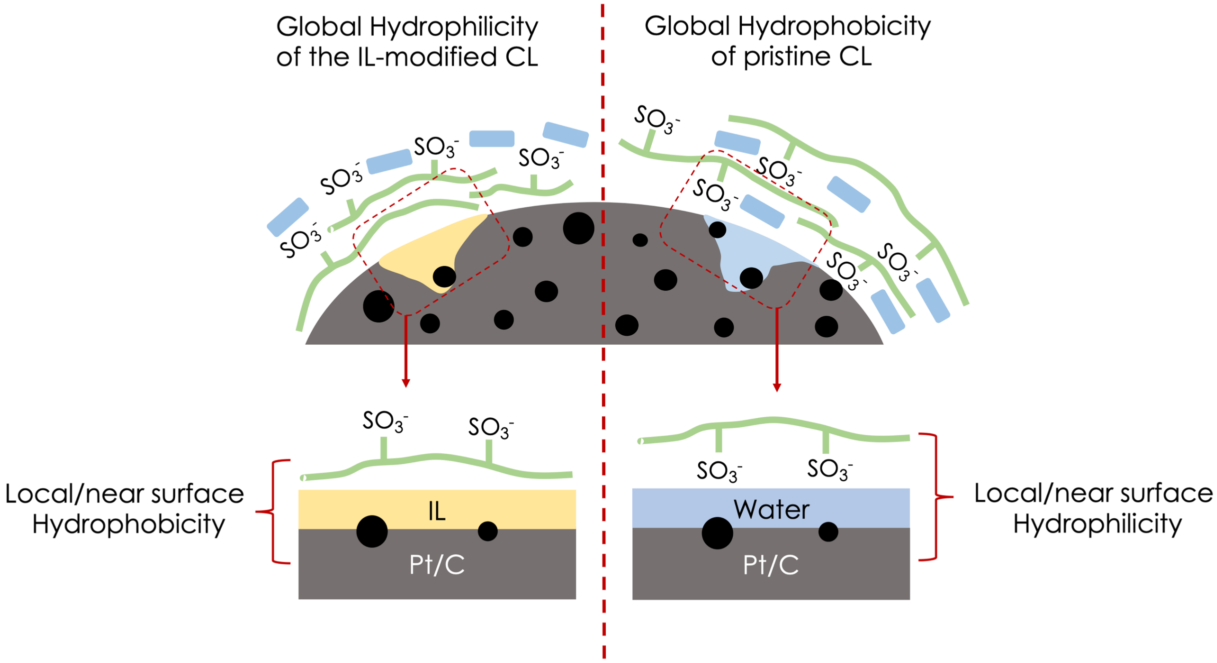

Figure S2. Nafion orientation near pores filled with IL vs. water.

Table S1. Zeta potentials measurements for pristine and IL-modified Pt/C with and without Nafion.

Sample	Zeta potential (mV)	Sample	Zeta potential (mV)
Pt/C	-10.9±7.2	Pt/C + Nafion	-34.2±11.3
Pt/C-($[C_2mim]^+[NTf_2]^-$)	-27.8±7.4	Pt/C-($[C_2mim]^+[NTf_2]^-$) + Nafion	-43.1±7.1
Pt/C-($[C_4mim]^+[NTf_2]^-$)	-13.4±5.6	Pt/C-($[C_4mim]^+[NTf_2]^-$) + Nafion	-33.2±12.4
Pt/C-($[C_4dmim]^+[NTf_2]^-$)	-29.7±7.1	Pt/C-($[C_4dmim]^+[NTf_2]^-$) + Nafion	-40.8±7.2

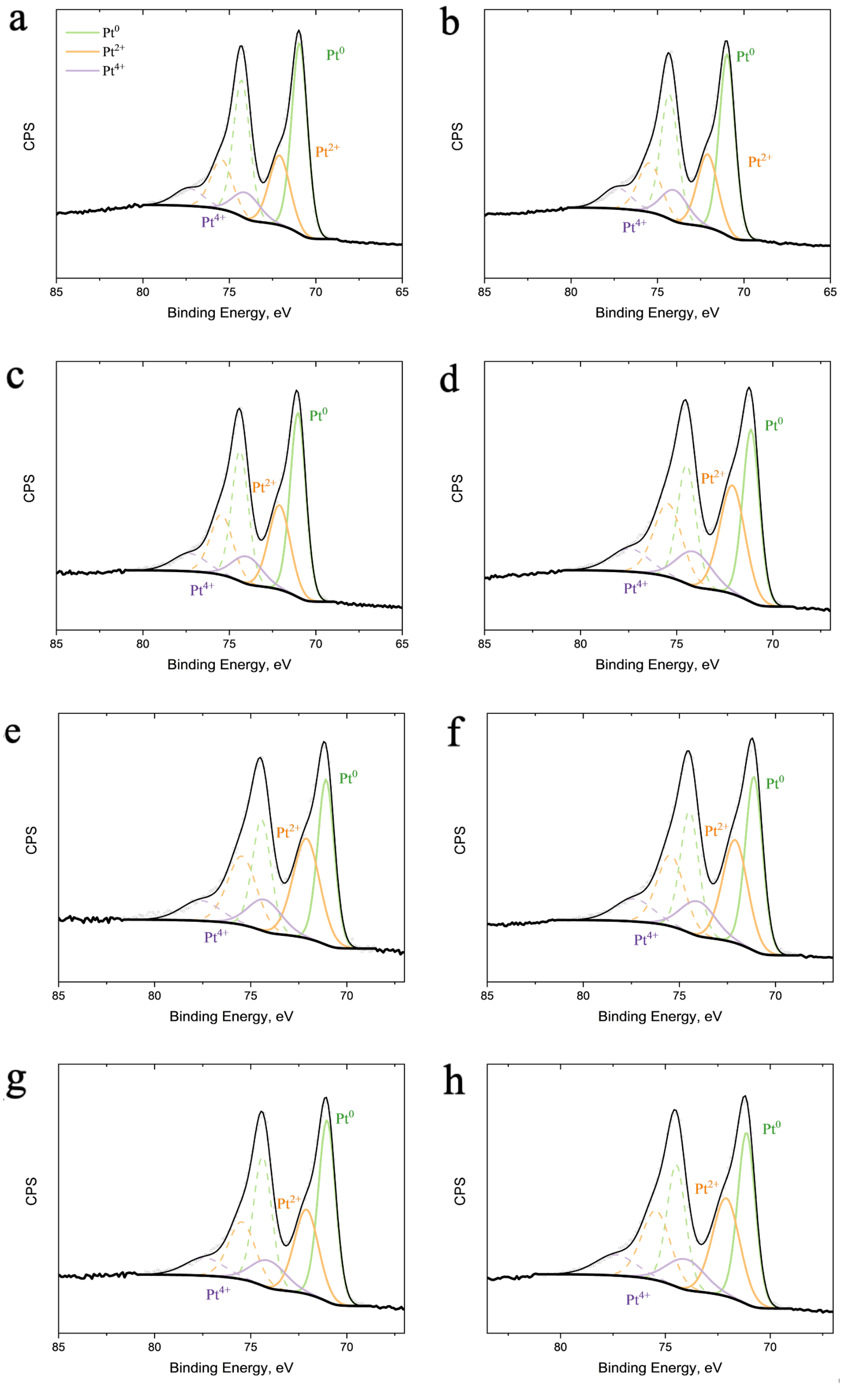

Figure S3. XPS Pt4f spectra of 40 wt. % Pt/C a) before CO introduction, b) after CO poisoning, IL-modified Pt/C catalysts including Pt/C-($[C_2mim]^+[NTf_2]^-$) c) before CO introduction, d) after CO poisoning, Pt/C-($[C_4mim]^+[NTf_2]^-$) e) before CO introduction, f) after CO poisoning, and Pt/C-($[C_4dmim]^+[NTf_2]^-$) g) before CO introduction, h) after CO poisoning.

Table S2. Pt and Pt-oxides binding energies before and after introduction of CO.

%At. Con. (St.Dev.)		Pt^0	Pt^{2+}	Pt^{4+}
**Baseline Pt/C**	Before CO introduction	58.6 (1.13)	28.1 (1.34)	13.3 (0.82)
	After CO poisoning	55.3 (1.22)	29.0 (1.25)	15.7 (0.81)
**Pt/C-($[C_2mim]^+[NTf_2]^-$)**	Before CO introduction	47.2 (4.66)	38.5 (6.86)	14.3 (2.61)
	After CO poisoning	44.4 (3.35)	38.1 (4.03)	17.5 (2.25)
**Pt/C-($[C_4mim]^+[NTf_2]^-$)**	Before CO introduction	52.5 (2.62)	32.97 (4.08)	14.57 (2.02)
	After CO poisoning	42.3 (3.08)	40.2 (4.02)	17.5 (2.13)
**Pt/C-($[C_4dmim]^+[NTf_2]^-$)**	Before CO introduction	51.4 (2.94)	33.5 (5.23)	15.1 (3.05)
	After CO poisoning	44.4 (3.40)	39.0 (4.06)	16.6 (2.13)

Table S3. Electrochemical properties of the Pt/C and Pt/C-IL samples evaluated by RDE.

	**Baseline Pt/C**	**Pt/C-($[C_2mim]^+[NTf_2]^-$)**	**Pt/C-($[C_4mim]^+[NTf_2]^-$)**	**Pt/C-($[C_4dmim]^+[NTf_2]^-$)**
**Mass Activity at 0.9 V ($A\ g^{-1}_{Pt}$)**	261±29.4	155±28.9	202±82.1	136±7.1
**Specific Activity at 0.9 V ($\mu A\ cm^2_{Pt}$)**	257±59.3	181±22.2	245±72.3	182±9.1
**ECSA ($m^2\ g^{-1}$)**	91.0±15.1	88.7±5.7	76.54±2.6	73.7±1.3
**R_H^+ ($\Omega\ cm^2$)**	3.16±0.09	0.85±0.64	0.90±0.49	2.21±0.56

Table S4. Electrochemical properties of Pt/C-IL MEAs containing various loadings of IL.

	IL/C=0	IL/C=0.64	IL/C=1.28	IL/C=2.56
**OCV (V)**	0.905	0.963	0.948	0.922
**Peak power density ($W\ cm^{-2}$)**	0.831	0.809	0.909	0.566
**Mass Activity at 0.9 V ($A\ g^{-1}_{Pt}$)**	288	173	347	110
**Specific Activity at 0.9 V ($\mu A\ cm^{2}_{Pt}$)**	399	509	697	491
**ECSA ($m^2\ g^{-1}$)**	72.1	60.6	49.7	22.4
**Local O_2 mass transport resistance ($s\ cm^{-1}$)**	0.175	0.256	0.268	0.258

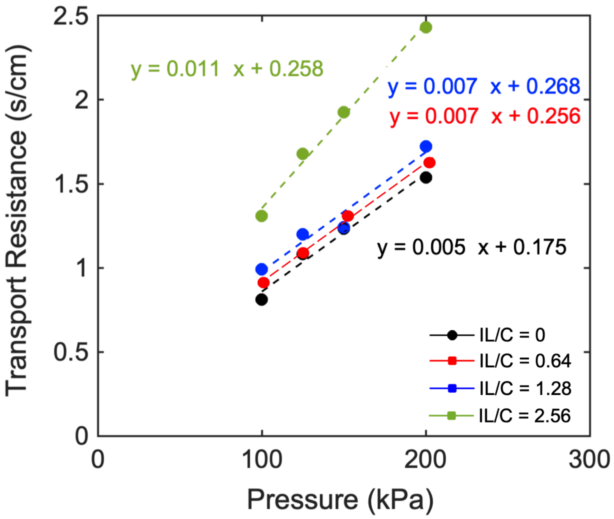

Figure S4. Mass transport resistance plots of Pt/C and Pt/C-($[C4mim]^{+}[NTf2]^{-}$) with various loadings of IL obtained at 75% RH, at 100, 125, 150, 200 kPa_{abs} total pressure in 5 cm^2 differential cells.

Evolution of Pt/C-IL interface during MEA fabrication procedure

100 mg of pristine Pt/C and all three Pt/C-IL powders were mixed with 2 ml of IPA and DI water and ultrasonicated for 30 mins. 100 mg of IL-impregnated powder with IL to C ration of 1.28 contains about 43.75 mg of IL. In the next step, we centrifuged the suspensions at 30000 rpm for 15 mins until the powder separated completely from the solvents as can be seen in Figure S6. Solvents were then removed from the centrifuge tubes and remaining powders dried overnight. The weight difference between the original IL-impregnated powders and the ones after the procedure can be attributed to the ILs that dissolved in the solution as all three ILs are soluble in the mixture of IPA and water.

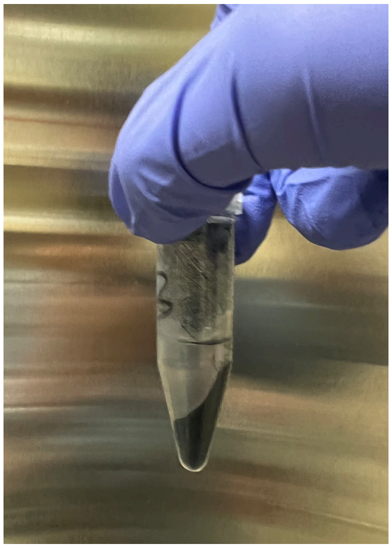

Figure S5. Separation of Pt/C-IL powder from solution after centrifuge.

This weight difference for Pt/C-($[C_2mim]^+[NTf_2]^-$), Pt/C-($[C_4mim]^+[NTf_2]^-$), and Pt/C-($[C_4dmim]^+[NTf_2]^-$) powders, were 18.1, 22.1, and 24.8 mg, respectively. This procedure was also performed on a control sample in which we saw about 10.6 mg weight loss. This loss is due to the potential error in the experiment as we remove the separated solution from the precipitated powder. Therefore, the weight of Pt/C-($[C_2mim]^+[NTf_2]^-$), Pt/C-($[C_4mim]^+[NTf_2]^-$), and Pt/C-($[C_4dmim]^+[NTf_2]^-$) powders decreased about 17, 26, and 32 wt.% that is attributed solely to the IL dissolved in the IPA and water mixture and got separated from the powder. This means that a part of the ILs in the impregnated powders redispersed in the solution and redeposited together with Nafion. We believe, IL molecules on catalyst surface, in the macropores and bigger mesopores would dissolve faster compared to the ones in the smaller mesopores and micropores. This observation is in great agreement with the IL molecules chemical structures and BET results. As can be seen, the ILs dissolution in IPA and water increases with elongation in alkyl chain length. For example, ($[C_4dmim]^+[NTf_2]^-$) showed the highest dissolution (about 30wt.%) in IPA and water and has the most

complex structure of all three ILs, as it has both butyl and methyl cationic chains, and less packing of the molecules in the pores. Therefore, the more complex the structure of IL is, the less it fills the pores and easily get redispersed in the solution.

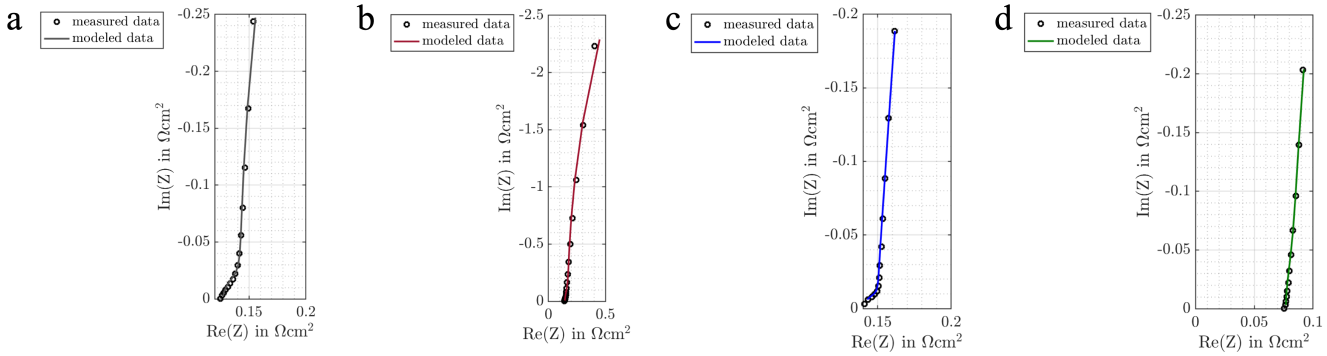

Figure S6. Nyquist plots measured at 80°C, 100% RH, and 100 kPa_{abs} total pressure in 5 cm^2 differential cells for a) baseline Pt/C, b) Pt/C-($[C_2mim]^+[NTf_2]^-$), c) Pt/C-($[C_4mim]^+[NTf_2]^-$), d) Pt/C-($[C_4dmim]^+$ $[NTf_2]^-$) MEAs.

.

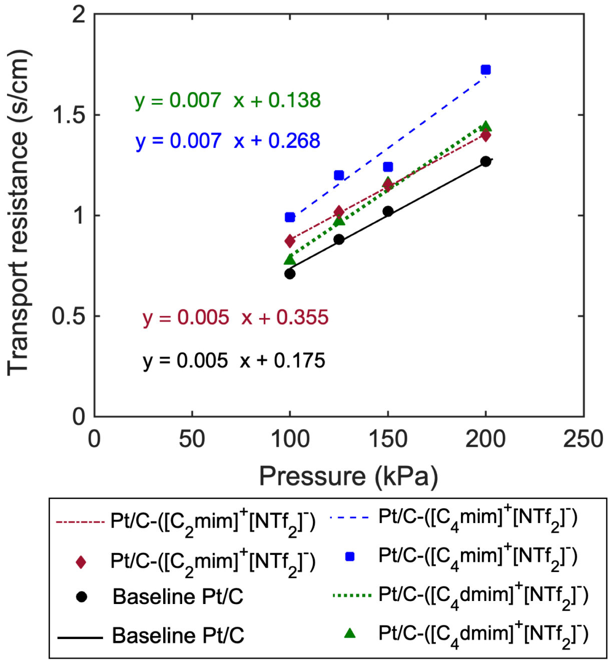

Figure S7. Mass transport resistance plots of Pt/C and Pt/C-IL MEAs obtained at 75% RH, at 100, 125, 150, 200 kPa_{abs} total pressure in 5 cm^2 differential cells.

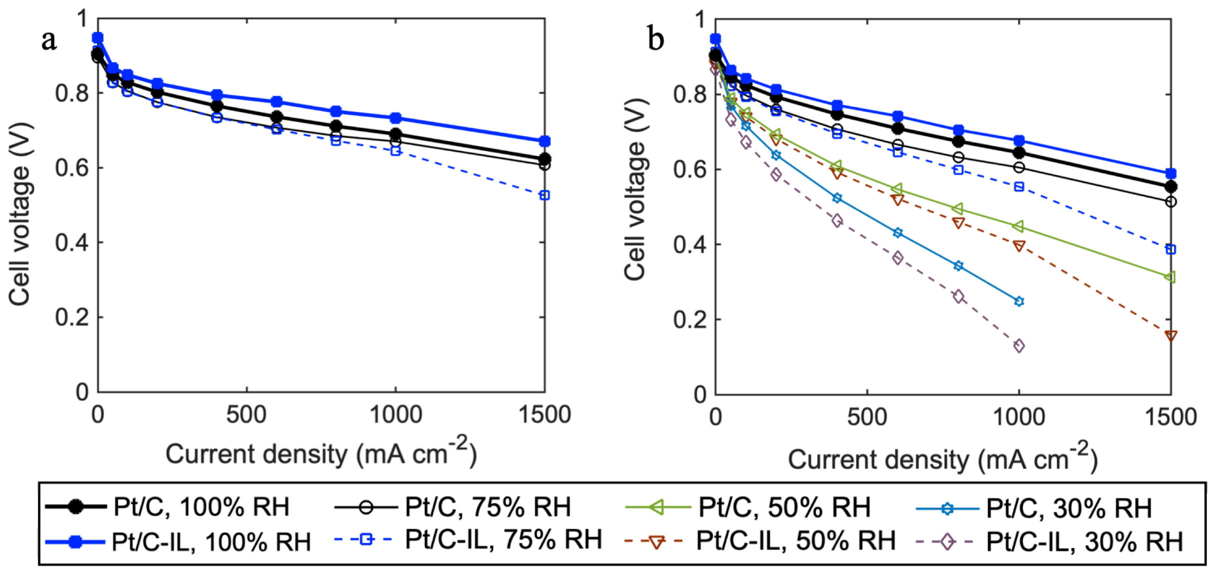

Figure S8. a) HFR-corrected H_2/air polarization curves of Pt/C and Pt/C-($[C_4mim]^+[NTf_2]^-$) at 75% and 100% RH and 150 kPa_{abs} total pressure, b) H_2/air polarization curves of Pt/C and Pt/C-($[C_4mim]^+[NTf_2]^-$) measured at 100%, 75%, 50%, and 30% RH in 5 cm^2 differential cells.

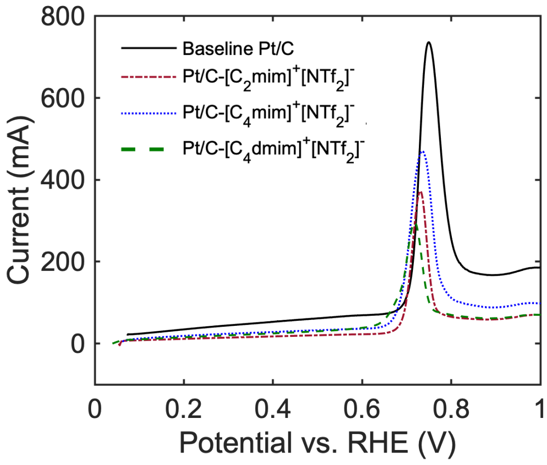

Figure S9. CO-stripping of Pt/C and Pt/C-IL MEAs.

Table S5. Electrochemical properties of Pt/C and Pt/C-IL MEAs after AST.

	Baseline Pt/C	Pt/C-($[C_2mim]^+[NTf_2]^-$)	Pt/C-($[C_4mim]^+[NTf_2]^-$)	Pt/C-($[C_4dmim]^+[NTf_2]^-$)
**Cell potential @ 0.8 A cm^{-2}**	0.361	0.495	0.595	0.479
**Peak power density**	0.349	0.474	0.642	0.460
**Mass Activity at 0.9 V (A g^{-1}_{Pt})**	21.6	15.1	29.17	7.83
**Specific Activity at 0.9 V (μA cm^{-2})**	163	196	165	40
**ECSA (m^2 g^{-1})**	1.32	7.69	17.63	19.45

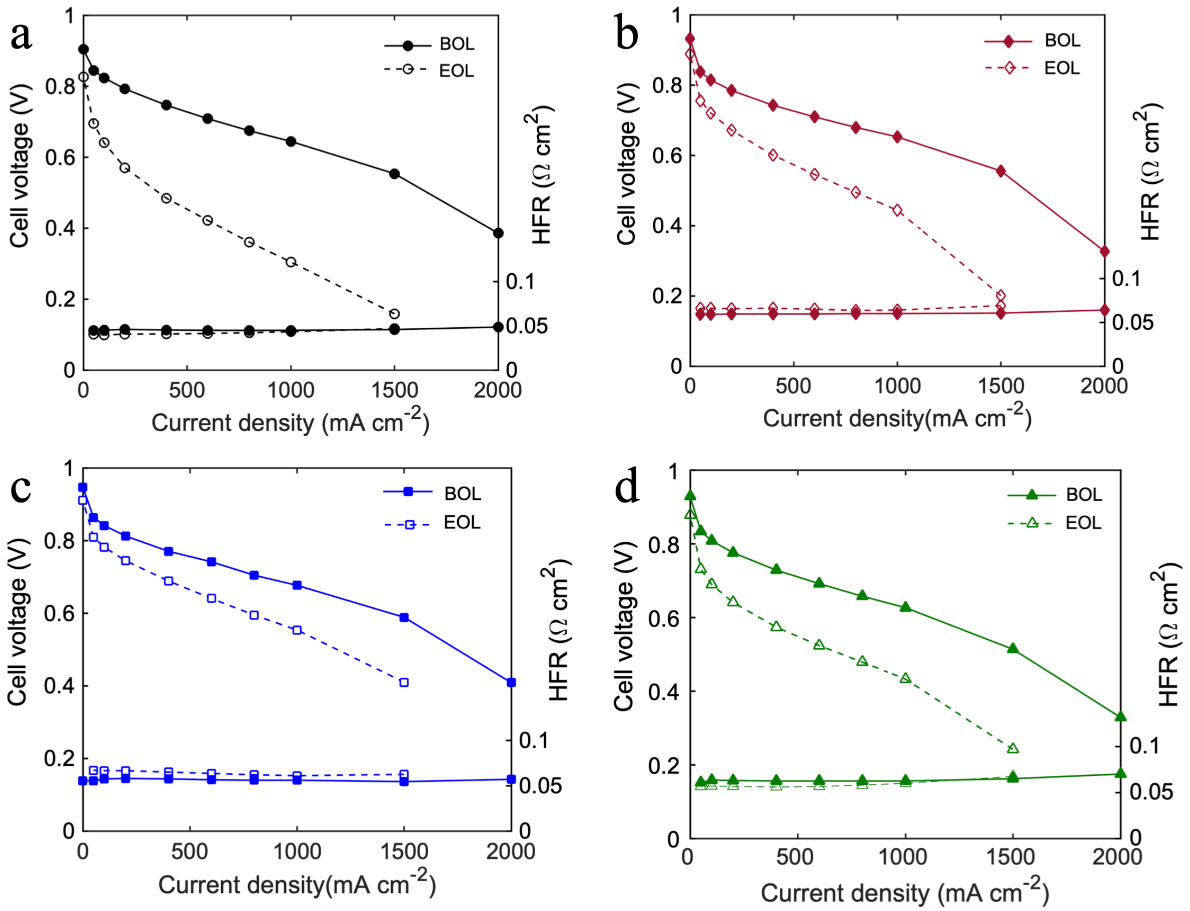

Figure S10. a) comparison of H_2/air polarization curves at the beginning of life and after 30,000 cycles for a) baseline Pt/C, b) Pt/C-($[C2mim]^+[NTf2]^-$), c) Pt/C-($[C4mim]^+[NTf2]^-$), d) Pt/C-($[C4dmim]^+$ $[NTf2]^-$) MEAs.

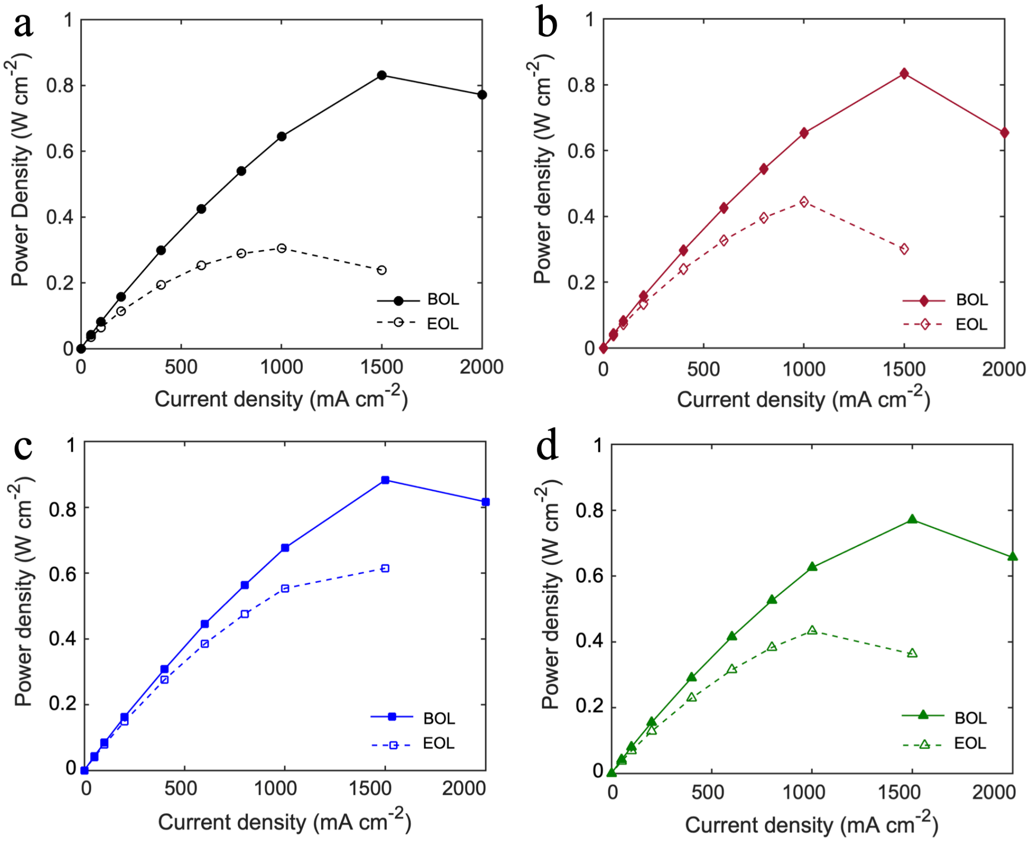

Figure S11. a) comparison of H_2/air peak power densities at the beginning of life and after 30,000 cycles for a) baseline Pt/C, b) Pt/C-($[C_2mim]^+[NTf_2]^-$), c) Pt/C-($[C_4mim]^+[NTf_2]^-$), d) Pt/C-($[C_4dmim]^+ [NTf_2]^-$) MEAs.
